# Adherence and factors affecting satisfaction in long-term telerehabilitation for patients with chronic obstructive pulmonary disease: a mixed methods study

**DOI:** 10.1186/s12911-016-0264-9

**Published:** 2016-02-25

**Authors:** Hanne Hoaas, Hege Kristin Andreassen, Linda Aarøen Lien, Audhild Hjalmarsen, Paolo Zanaboni

**Affiliations:** Norwegian Centre for E-health Research, University Hospital of North Norway, P.b 35, 9038 Tromsø, Norway; Faculty of Health Science, Department of Clinical Medicine, UiT The Arctic University of Norway, P.b 6050, Langnes, Tromsø, 9037 Norway; LHL- klinikkene Skibotn, 9143 Skibotn, Norway; Department of Clinical Medicine, University Hospital of North Norway, Tromsø, Norway

**Keywords:** Adherence, Chronic obstructive pulmonary disease, Exercise, Pulmonary rehabilitation, Self-management, Telemedicine, Telerehabilitation

## Abstract

**Background:**

Telemedicine may increase accessibility to pulmonary rehabilitation in chronic obstructive pulmonary disease (COPD), thus enhancing long-term exercise maintenance. We aimed to explore COPD patients’ adherence and experiences in long-term telerehabilitation to understand factors affecting satisfaction and potential for service improvements.

**Methods:**

A two-year pilot study with 10 patients with COPD was conducted. The intervention included treadmill exercise training at home and a webpage for telemonitoring and self-management combined with weekly videoconferencing sessions with a physiotherapist. We conducted four separate series of data collection. Adherence was measured in terms of frequency of registrations on the webpage. Factors affecting satisfaction and adherence, together with potential for service improvements, were explored through two semi-structured focus groups and an individual open-ended questionnaire. Qualitative data were analysed by systematic text condensation. User friendliness was measured by the means of a usability questionnaire.

**Results:**

On average, participants registered 3.0 symptom reports/week in a web-based diary and 1.7 training sessions/week. Adherence rate decreased during the second year. Four major themes regarding factors affecting satisfaction, adherence and potential improvements of the intervention emerged: (i) experienced health benefits; (ii) increased self-efficacy and independence; and (iii) emotional safety due to regular meetings and access to special competence; (iv) maintenance of motivation. Participants were generally highly satisfied with the technical components of the telerehabilitation intervention.

**Conclusions:**

Long-term adherence to telerehabilitation in COPD was maintained for a two-year period. Satisfaction was supported by experienced health benefits, self-efficacy, and emotional safety. Maintenance of motivation was a challenge and might have affected long-term adherence. Four key factors of potential improvements in long-term telerehabilitation were identified: (i) adherence to different components of the telerehabilitation intervention is dependent on the level of focus provided by the health personnel involved; (ii) the potential for regularity that lies within the technology should be exploited to avoid relapses after vacation; (iii) motivation might be increased by tailoring individual consultations to support experiences of good health and meet individual goals and motivational strategies; (iv) interactive functionalities or gaming tools might provide peer-support, peer-modelling and enhance motivation.

## Background

Pulmonary rehabilitation (PR) is a well-documented component in the management of chronic obstructive pulmonary disease (COPD) [1]. The main goal of PR is to improve the patient’s overall condition, both physically and psychologically, and to promote long-term adherence of health-enhancing behaviour [2]. PR has proven effective in reducing dyspnoea and improving functional exercise capacity and quality of life [1, 2]. PR is a comprehensive intervention, which includes patient assessment, patient-tailored therapies, such as education, behaviour change, and self-management support [2]. Exercise training is a cornerstone of a PR program [2, 3]. Without any maintenance strategy, benefits after PR usually diminish after 6–12 months. Quality of life is reported better maintained than exercise capacity [2].

Available resources for PR vary among different health care settings and might affect attendance and adherence [2, 4]. Patients living in rural areas especially suffer from poor availability of PR programs [5, 6]. In Norway, specialist competence in PR in community settings is lower than in England and the Netherlands [7], and even fewer exercise maintenance programs are available for patients with COPD, especially in rural areas like Northern Norway. Attendance rate to PR is also negatively affected, as up to 50 % of those patients who are offered PR decide not to attend [2]. Barriers, which prevent patients from attending PR, include disruption of their everyday routine, travel or transportation difficulties, inconvenient timing of the program, lack of perceived benefit, lack of social support, low self-confidence, and fear of being breathless or exacerbating existing medical problems [2, 8–10]. High dropout rates are an additional challenge in PR. Dropout rates vary between studies, ranging from 10 % to 32 % [2]. A study investigating attendance and adherence by COPD patients to PR programs found that 29.1 % of the patients attending PR participated in less than 63 % of the sessions, and were categorised as "non-adherers". The study concluded that age, smoking status, availability of social support, travel distance, and markers of disease severity, in particular use of long-term oxygen therapy, were strong predictors of attendance and adherence to PR [11]. A qualitative study exploring the perspectives of patients with COPD on adherence to PR suggested that adherence could be enhanced by building confidence in the patient, fostering tangible results, and recognising and responding to the patient readiness and access issues [12].

Development of telemedicine-based rehabilitation interventions for patients with COPD attempts to meet the need for increased applicability and accessibility of PR. Telerehabilitation is defined as the delivery of medical rehabilitation service at a distance, regardless of the patients’ geographical location, using electronic information and communication technologies [13]. This often implies two-way video communication between the provider and the patient [13]. In contrast to conventional PR, which is often offered as shorter, intensive programs, telerehabilitation has the potential to provide long-term follow-up in the patients’ homes. Telerehabilitation for patients with COPD often includes exercise and/or self-management education [14–17]. In addition, a telemonitoring component, which focuses on disease management and monitoring of symptoms, for example after an exacerbation or hospitalisation, may be included [18].

There is still little evidence of the benefits of telemedicine in PR [2]. A recent review showed that telerehabilitation may lead to increased physical activity level, but no effect was detected on exercise capacity and dyspnoea [19]. Other studies have shown promising results for telerehabilitation for patients with COPD in terms of feasibility, safety, exercise capacity, physical activity, health-related quality of life, and reduced hospital admissions [14–16, 20–22]. Dropout rates vary greatly between these studies, ranging from 0 % to 45 % [14–16, 19–22]. Information about adherence to telerehabilitation for patients with COPD is limited. A pilot study of 4-week telerehabilitation intervention showed good adherence by patients to wear a device for ambulant activity monitoring and real-time coaching of daily activity behaviour and to fill in a web-based diary (median use was 109 % and 58 % of the recommended times, respectively) [15]. In another 9-month pilot study, the median use of a web-based triage diary was 82.8 % and the median adherence to prescribed exercises was 21.0 % [16]. Adherence seems to vary just as much between patients as between different telerehabilitation interventions. To our knowledge, no criteria for stratification of patients with COPD who would benefit from telerehabilitation have been developed yet.

Program effectiveness depends on adherence. Adherence to conventional rehabilitation programs and telemedicine interventions may be influenced by patients’ satisfaction [19, 23]. Thus, it is important to understand factors affecting satisfaction when planning and developing new telerehabilitation interventions. Together with other patient-related outcomes, patients’ satisfaction, perceived usability of the technology, and adherence represent crucial factors to consider when implementing telerehabilitation interventions in routine use for patients with COPD. However, patients’ perspectives in telerehabilitation are sparsely documented. We have found only one recent study exploring in depth the attitudes of patients with COPD towards telerehabilitation [24]. Others reported that patients were generally satisfied with telerehabilitation solutions, but experienced some technological usability problems [15, 16].

We conducted a two-year pilot study on telerehabilitation in COPD. Clinical and other patient-reported outcomes have been reported elsewhere [14, 25]. The aim of the current study was to explore the patients’ perspectives in long-term telerehabilitation in COPD. We focused our study on adherence and patients' experiences, aiming to identify factors affecting satisfaction and potential for improvements that might increase adherence. Our research questions were: i) How well do participants adhere to telerehabilitation in COPD? ii) Which are the main factors affecting satisfaction and adherence to long-term telerehabilitation program for patients with COPD? iii) Which are the potential improvements that might increase adherence?

### Theoretical framework

The theoretical background used in this study was based on selected theories that are suited to highlight patients’ perspectives and address the study objectives.

Our work was empirically based, but the analysis was theoretically informed. Theories of health promotion and self-efficacy provided some key concepts [26, 27]. Further, our analysis was based on a view of health as “the ability to adapt and self-manage in the face of social, physical, and emotional challenges” [28]. This dynamic view of health emphasises the individual’s resilience or capacity to cope, maintain or restore one’s integrity, equilibrium, and sense of wellbeing as one face the many challenges of life. This view of health is also in line with Antonovsky’s salutogenic theory [29]. Interpretations were informed by Bordin’s definition of therapeutic alliance, which consists of three elements: an agreement on the goals of the treatment, agreement on the tasks and methods, and the development of a personal bond between the person seeking change and the helper (therapist) [30]. Motivational strategies were analysed in the light of regulatory focus theory. According to the regulatory focus theory, motivational strategies can be guided by a promotion or a prevention focus [31]. A promotion focus aims to accomplish positive outcomes, while a prevention focus aims to avoid negative outcomes. Individuals with a promotion focus will therefore tend to get motivated by accomplishments, hopes, and aspirations, while individuals with a prevention focus are more likely to get motivated by safety, responsibilities, and obligations [31].

## Methods

### Study description and intervention

We conducted a pilot study of a two-year telerehabilitation intervention for patients with COPD [14, 25]. Telerehabilitation consisted of three components: exercise training at home, telemonitoring and self-management. Exercise training was performed by participants on a treadmill at home. Participants received an individually tailored interval-training program, consisting of a warm-up period followed by 4 bouts up to 4 min of walking at a higher intensity alternated by bouts of lower intensity or full stop. The program ended in a cool-down period. Training was recommended three times a week, and the duration of each session was ≥30 min. Perceived intensity to be aimed for during interval bouts was 6 on the Borg CR10 scale, corresponding to a strenuous exercise level [32, 33]. Participants were provided with a tablet computer (Apple iPad 2) and a pulse oximeter used for telemonitoring. Videoconferencing sessions up to 30 min between participant and tele-physiotherapist were performed weekly. The tablet computer was also used to access a webpage for self-management which included: an individual training program, a daily diary for reporting symptoms and oxygen saturation at rest, a training diary for reporting exercise duration, perceived exertion, oxygen saturation and heart rate during exercise, and historical data. Data were also accessible to the tele-physiotherapist and used for discussion with the patients.

The Regional Committee for Medical and Health Research Ethics, North Norway, approved the study, and all participants signed a written consent form for the study.

### Participants

Among patients completing a four-week inpatient pulmonary rehabilitation program, the physician in charge selected eligible patients with COPD in a stable health condition and deemed physically capable to perform safely exercise at home. Patients were then invited to take part to the study, and participation was on a voluntary basis. Ten patients with moderate to severe COPD were recruited for the pilot study. The average age of participants was 55.2 years. Eight participants were retired. Five lived with their spouses or other family members. Half of the participants were women. Three used long-term oxygen therapy. Average travel distance to the closest hospital was 99 ± 76 km (range 3–218 km). Eight participants used the Internet daily or nearly every day before the intervention, while two were inexperienced computer users.

### Triangulation of data collection techniques

A mixed methods approach and a triangulation of data collection techniques were selected to address the study objectives. Four methods for data collection were utilised to explore our research questions. Adherence to the telerehabilitation intervention was measured through the analysis of logs on the webpage. Factors affecting satisfaction and adherence, together with potential for service improvements, were explored through focus groups and individual open-ended questionnaires. User-friendliness and technical improvements were examined through a standardised questionnaire and open-ended questionnaires.

### Analysis of logs

Adherence to the telerehabilitation intervention was assessed by the dropout rate and adherence rate. A dropout was defined as a participant failing to participate to the intervention as required by the study protocol. The dropout rate was therefore calculated by the percentage of participants who did not attend the final visit and were no longer active. Adherence was defined in relation to what extent the participants used the intervention compared to the recommendations [34]. Logs on the webpage were extracted to measure, for each participant, the average number of daily diary registrations per week and the average number of training sessions per week. Recommendation regarding daily diary registrations and training sessions was 7 times per week and 3 times per week, respectively. The adherence rate for the daily diary was measured by the number of registrations entered divided by the number of those recommended (7 times per week). The adherence rate for the training diary was measured by the number of training sessions performed divided by the number of those recommended (3 times per week). Descriptive statistics were summarised for the whole study period and stratified for the first and the second year of follow-up. Diary registrations and training sessions were also analysed by 3-month intervals to show the trend from baseline to study end. Since the 10 participants started at different times, the average use for each month of the year was also computed to detect whether there was any seasonality. Finally, the average for each participant was summarised to show individual differences in adherence.

### Focus groups

We relied our study of satisfaction and potential improvements on focus groups because we wanted to open the analysis for new angles and connections to everyday life from the participants' view. Focus groups are used to benefit from the group interaction to produce data and insight [35]. We used the focus groups to explore the participants’ experiences with the intervention and technology and to facilitate a creative process between the participants and the research team. The goal was to explore factors affecting satisfaction and discover potential improvements which might affect adherence.

Two semi-structured focus groups took place during a two-day meeting in June 2013 and May 2014 at the rehabilitation centre where the participants were recruited. All 10 participants took part in at least one of the two focus groups. One moderator (P.Z.) and one co-moderator (L.A.L.) led the first focus group. This was focused mainly on user-friendliness and experiences with the technological equipment. The second focus group, led by one moderator (H.H.) and two co-moderators (L.A.L. and P.Z.), focused on experiences with participating in a long-term telerehabilitation intervention and potential for service improvements. H.H. knew some of the participants from a previous work relationship at the rehabilitation centre. L.A.L. was the physiotherapist supervising the participants via telemedicine. P.Z. did not have any relationship with the participants beforehand. A semi-structured interview guide was used. The moderators presented topics using open-ended questions, facilitated the dialogue among the participants and followed up with further questions in an informal and open atmosphere. The participants allowed themselves to discuss relatively freely about their experiences. They mirrored each other, discussed different opinions, and presented new topics. The moderators sought to summarise discussions around the various topics consecutively, so that interpretations of the informants’ answers were verified directly. Both focus groups lasted for 1.5 h and were recorded. Recordings were transcribed verbatim in Norwegian. Quotes used in this paper were later translated into English.

### Open-ended questionnaires

An individual open-ended questionnaire focused on personal experiences, benefits and challenges in participating in long-term telerehabilitation was collected and analysed together with the transcripts from the focus groups. The questionnaires were collected for all patients after the first and the second year of participation, just before the focus groups.

### User-friendliness questionnaire

The System Usability Scale (SUS) is a questionnaire used to grade the usability of systems [36]. SUS is considered a valid and reliable tool for evaluation of a wide variety of products and services, including mobile devices and webpages [37]. It consists of a 10-item questionnaire with five response options (5-point Likert scale with anchors for Strongly agree and Strongly disagree). SUS results yield a score between 0 and 100, with 100 indicating best usability. In the current study, SUS was used to measure user-friendliness of the telerehabilitation intervention [36], and was completed by the participants at the end of the first year, before conduction of the first focus group.

### Data analysis

**Fig. 1:**
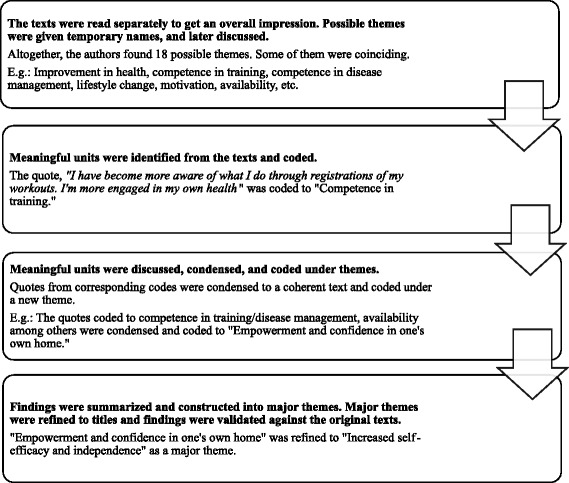
The steps of analysis in systematic text condensation with examples

Analysis of the qualitative data focused on identifying topics rather than on differences and similarities between individual respondents, thus confirming to a thematic analysis technique [38]. Further, the material was analysed using systematic text condensation, which is inspired by Giorgi and modified by Malterud [39, 40]. In the current study, three of the authors read the material separately to get an overall impression, which was later discussed. Secondly, the text was analysed individually in search for meaning units that could represent the participants’ experiences and adherence to the intervention. Then these meaning units were discussed, condensed and coded under themes. In the end, findings were summarised and constructed into major themes that could represent factors affecting satisfaction and adherence, or potential for service improvement (Fig. 1).

## Results

### Adherence

On average, the participants were in the study for 740 days. No dropout occurred. They all participated actively during the two-year period and attended all planned visits at baseline, 1 year and 2 years. The participants experienced some relapses due to different reasons, including holidays, traveling, sickness, or hospital admissions. However, they all rejoined the program after these breaks. One participant stopped registering in the daily diary and training sessions two months before the study end, but still maintained regular contacts with the tele-physiotherapist during this period.

On average, participants registered 3.0 diary registrations/week and 1.7 training sessions/week via the webpage during the whole study period. This corresponds to an average adherence rate of 43.3 % and 56.2 %, respectively (Table 1). Use and adherence decreased from the first to the second year. On average, participants had 3.4 diary registrations/week and 2.1 training sessions/week in the first year, and 2.6 diary registrations/week and 1.2 training sessions/week in the second year.

**Table 1:** Average adherence to the daily diary and training sessions during the study

N = 10	Average year 1	Adherence year 1	Average year 2	Adherence year 2	Average total	Adherence total
Daily diary registrations	3.4/week	48.5 %	2.6/week	37.0 %	3.0/week	43.3 %
Training sessions	2.1/week	69.1 %	1.2/week	40.5 %	1.7/week	56.2 %

**Fig. 2:**
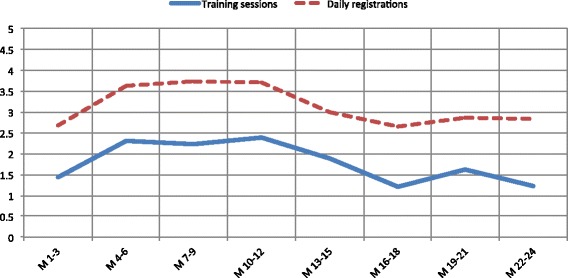
Monthly adherence rate for training sessions and diary registrations with three-month intervals

Adherence rate for training sessions increased during the first three months of the study to a level close to the recommendation (Fig. 2). Adherence was then maintained during the first year, while it decreased from the second year until the study end. Adherence rate for diary registrations showed a similar trend, but with lower variations over the two-year period (Fig. 2).

**Fig. 3:**
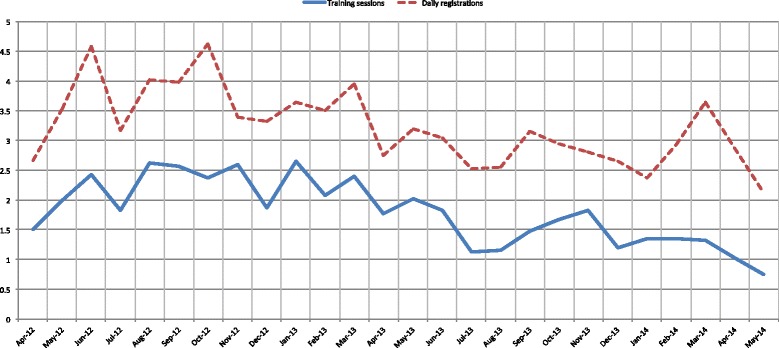
Seasonality of adherence rate for training sessions and diary registrations

Figure 3 shows the seasonality of training sessions and diary registrations. There were some negative troughs during December (Christmas) and July-August (summer holidays) during which participants performed fewer training sessions. The tele-physiotherapist encouraged the participants to take time-off during holidays, underpinning exercise as an ordinary job. Diary registrations appear to be affected by the same seasonality as training sessions (Fig. 3).

Table 2 summarises the individual adherence rates for the ten participants for the two-year period. Adherence to the daily diary ranged from 8.2 % to 98.3 %, while adherence to training session ranged from 16.3 % to 99.1 %.

**Table 2:** Adherence, diary registrations and training sessions per participant during the study

Participant n = 10	Treatment days	# Diary registrations	# Training sessions	Diary reg/week	Training/week	Adherence to diary registrations in %	Adherence to training sessions in %
F 1	780	593	329	5.3	3.0	76.0	98.4
M 2	765	752	325	6.9	3.0	98.3	99.1
M 3	704	540	124	5.4	1.2	76.7	41.1
M 4	753	133	128	1.2	1.2	17.7	39.7
F 5	752	185	83	1.7	0.8	24.6	25.8
M 6	752	218	219	2.0	2.0	29.0	68.0
F 7	743	61	52	0.6	0.5	8.2	16.3
F 8	704	275	171	2.7	1.7	39.1	56.7
F 9	709	189	109	1.9	1.1	26.7	35.9
M 10	738	273	258	2.6	2.4	37.0	81.6
Average	740	321.9	179.8	3.0	1.7	43.3	56.2

M = Male. F = Female. Recommendations for diary registrations and training sessions were respectively 7 and 3 times/week

### Factors affecting satisfaction and adherence

Four major themes regarding factors affecting satisfaction and adherence, and potential for service improvement emerged from the analysis of the data collected: (i) experienced health benefits; (ii) increased self-efficacy and independence; (iii) emotional safety due to regular meetings and access to special competence; and (iv) maintenance of motivation. Quotes from the participants used to highlight the themes, are marked with participant number and sex (F for female and M for male).

#### Experienced health benefits

A positive change in experienced level of health throughout the two-year period was mentioned by all the participants. Experienced health benefits were interpreted as one of the main factors affecting satisfaction. The second focus group started with the question: “What do you think about participating in the study?” The first reply was as follows:

*Gold and green forests… in terms of my health… compared to before. It has been effective, even though you are plagued with pneumonia and all that, which drags you down, but after a while, you get back on your feet again, up, on your own peak. It is not much, but I have to call it my own peak* (M10).

Despite having a chronic disease, the participants felt in good health most of the time. They described a broad perspective of health and health benefits consequent to their participation in the study, including physical, psychological, and social achievements. They also valued these benefits in relation with their everyday life. Some of the most colourful and descriptive statements regarding experienced health benefits appeared on the individual open-ended questionnaires after the first year:

*The study gave me better health and immune defence. Now it is easier to cope with problems that are there almost all the time when you have COPD and just 30 % of your lung capacity left. The spark of life is increasing. This means that you meet your days easier from morning to night* (M10).

*I can tag along with my family on trips, and go fishing with my grandchildren* (M4).

*My couch is less worn down than it would be without this project* (M3).

#### Increased self-efficacy and independence

The participants proudly described an increased competence in a variety of different fields. These mastery experiences seemed to have created a higher degree of self-efficacy and confidence in self-management of their exercise regime, everyday life, and disease.

An increased competence and reflection on how they best can adjust and perform their exercise regime can be exemplified in the following quotations:

*There are many different elements that might affect how you complete the exercise and what you think about it* (M4). *-Yes, like the temperature in the room, which other activities you have been doing that day, if you exercise in the morning or in the evening* (M6).

Mastery experiences in training influenced also their independence and self-efficacy in other areas of their everyday life:

*I have become stronger mentally. When I managed something physically, I somehow got stronger mentally as well. I felt: Yes! I managed to do the housework, and then I dared to go to the grocery store as well* (F1).

Curiosity, access to a pulse oximeter and a mastery experience from the everyday life improved one of the participants’ self-management of her disease:

*I saw the light one day. I was using the oximeter while cleaning the house, and I discovered that I was sooo low. I didn’t use the oxygen while doing housework before, but I do now* (F8).

For many of the participants, self-efficacy seemed somehow connected to acceptance and control of their disease. In one section of the second focus group, they talked about the shame and stigma they might feel while being out in the society:

*I felt ashamed to ask the woman at the cashier for help… I felt shame, but then I thought: Why bother with the shame?! If you need help, you need help. That’s a done deal. Then I felt better. Now I can ignore other people. I have to focus on myself to be able to go through the shopping mall* (M2).

*You have to accept your disease. When you have done everything you can to be at your best health, when you have exercised as much as you can, and things cannot get better, then you do not feel as ashamed any more. You actually do make an effort to keep on your own feet* (M8).

All participants were satisfied with the opportunity to exercise independently at home. In Northern Norway, where the study was conducted, there can be long travel distances to training facilities and harsh weather conditions, especially during winter. The availability of a treadmill at home appeared as a solution to these barriers and participants valued the broadened accessibility and availability: *You can register at any time of the day and we have even talked when I was in Turkey* (F8). In addition, the participants appreciated the possibility to exercise effectively at home thus avoiding the stigma of being observed while exercising:

*If I want to go to the gym, it is a 60 km drive from my house. Moreover, I would have felt weak in front of others. They would have looked at me, and thought: He cannot do anything. Then I would have felt it myself as well. I think I would have quit sooner* (M2). *–Kind of the opposite experience, but I got a lot of positive, but for me, unpleasant attention at the gym, especially since I got this oxygen tube in my nose. I heard like thirty times in one session: You are doing sooo good! Too much attention, well meant, but for me it was negative* (F8). *-I really enjoy going to the basement at home to exercise. There I can be alone, listen to music, and just walk. Then I actually can relax* (M2).

More independence was also described in the relationship with the therapist. With laughter, the participants reflected on how the technology made it easier for them to withdraw themselves without confrontation from the collaboration with the tele-physiotherapist. Through onomatopoeic words and statements like: *Bang!* (Referred to slamming the iPad cover together to break contact) and *Tut tut tut tut! No longer online*…, they described how they could become "unavailable" or "lose network access" if the follow-up did not “fit” their goals, mood or values, or if the relationship felt too challenging.

The nature of the telerehabilitation intervention, which encouraged daily registrations of symptoms and training sessions on the webpage, gave some of the participants increased control over their own health:

*I have become more aware of what I do through registrations of my workouts. I am more engaged in my own health* (F1).

Participants highlighted also that the intervention helped them establish *regular routines for exercising (M2 & M3).* However, one participant reported difficulties in performing regularly all the required activities: *I exercise, but I always forget to report the sessions (F7).*

#### Emotional safety due to regular meetings and access to special competence

The participants expressed that they felt safe and well taken care of in the study:

*It was comforting that the physio was there [over videoconferencing] when I started. She confirmed that I was doing it right. I think it is important that you do not go there by yourself and exercise in the wrong way. Then you knew you were on safe ground at least* (M6).

The tele-physiotherapists’ competence and experience with pulmonary diseases available via videoconferencing was particularly appreciated:

*My physio at home does not have the special knowledge about COPD. Pulmonary diseases are not her specialty field* (F8).

Participants valued their relationship with the tele-physiotherapist and the regularity of the videoconferences. These were seen as important factors for exercise maintenance:

*If the health personnel do not have knowledge about the disease, then it is a problem. However, they also have to know the person who has it* (M2).

*One of the best things with the project has been to meet the [tele-]physiotherapist once a week, and get to ask questions about everything that is on your mind* (F1).

#### Maintenance of motivation

After the first year, only two participants reported that their motivation was declining. Four participants described themselves as still highly motivated. The others described their motivation for exercising as good. The description of their level of motivation was more nuanced at the end of the second year. The participants felt that it was more difficult to start exercising again after periods of vacation and sickness. Seven of the participants stated that they had several periods with pneumonia or exacerbations during the second year. Two participants expressed lack of motivation and depression as reasons for not exercising or registering in the diary. Only one participant claimed to be highly motivated throughout the two years. Hence, maintenance of motivation was seen as a highly relevant challenge in this long-term telerehabilitation intervention. One of the participants described the increasing lack of motivation as follows:

*Everything went well during the first six months. I felt I increased my capacity every week. Then it stopped. Actually, I felt that my capacity started to decrease, and exercise became tougher to complete. I felt pleasure by the improvements. However, when it worsened, I struggled. I postponed and postponed, to the end of the day. This “upward feeling” is important, at least for me* (M6).

An increase in physical performance seemed to represent one of the main factors affecting motivation. However, this group of patients cannot increase their physical capacity infinitely. One of the participants, who adhered very well to the study, was able to set new goals when the “upward feeling” was lost:

*I could not run faster, but I could increase the duration and walk for a longer time. Then I felt an accomplishment* (F1)!

During the study period, the exercise program was progressed or changed by the tele-physiotherapist according to the participants’ registrations of the Borg CR10 scale for perceived exertion [32, 33], heart frequency, and oxygen saturation during interval peaks. The previous programs were not saved in the system. The participants experienced self-reported changes in physical capacity, especially related to better management of everyday life. However, they missed a more objective measure of exercise progress. As one stated and others supported:

*I would like to try the first exercise program I got. I remember how I struggled. I would like to try this now to see if there is any improvement* (F1).

Objective reports from graphs of the training diary and the daily diary that tracked changes in symptoms and exercise performance, seemed to be appreciated by the participants. Participants considered these graphs as motivational factors and learning opportunities.

In the first focus group, the participants mentioned how being part of a research study motivated them. Affiliation, being part of a “bigger” context and a sense of doing something for other patients with COPD, were motivational factors.

During the second year, the participants seemed to have entered a maintenance phase. They expressed that they were generally satisfied with life and had accepted their condition. They coped well with everyday life according to the varying symptoms of the disease:

*I organise my life in a different way now. I know my limitations and my physical capacity better (*M3).

When we searched the material for motivational strategies, we found that the participants used both a promotion and a prevention focus. Most of the participants distinctly reported a promotion focus related to motivation:

*Suddenly I could walk outside for 3 km quite fast without any stops. I also got comments on how good I looked. I have lost 10 kg* (F1).

However, we also find 2–3 participants using a prevention focus for describing their motivational strategy:

*My father had COPD. He just sat there, crying for the entire day, feeling sorry for himself. I can see him before me, and then I want to exercise. I don’t want to be like him! I don’t want to sit like that. What horror! This motivates me* (M2).

There was no connection between high adherence and a specific motivational strategy. The two participants with the highest degree of adherence expressed different motivational focuses.

### User-friendliness and technical improvements

The average score in the SUS was 83.5 (min 32.5, max 100). This indicates that the participants were highly satisfied with the equipment and considered it user-friendly.

The experience with the computer technology ranged from:

*After two years, I’m still not fluent with the iPad* (M4) to *In the beginning, I almost didn’t dare to touch the iPad, but now it is absolutely essential for me* (F1), and, *I had no experience with computers, but if I can learn this, everybody else can* (M2).

The participants had different suggestions for technical improvements of the intervention. Their main complaint was the stability of the videoconference connection. Technology that does not function the way it is supposed to is experienced as stressful. As one participant stated*:*

*I had many difficulties during videoconference with the physiotherapist. Sometimes we saw each other, but could not speak. Sometimes we could speak, but we could not see each other. It has been a slight nightmare at times* (M3).

Other suggestions for improvements were: more flexible exercise programs that could take into account “good days and bad days,” bigger buttons in the webpage, more training in the use of the tablet and the webpage, including interpretations of the diary and exercise registrations. The reliability of the measurements from the pulse oximeter was brought forward as important for completing and keep up with the registrations. Finally, participants would have preferred to meet face to face more often to discuss experiences and challenges. Some saw a potential in meeting in a group videoconference, but the strongest opinion was that they would benefit more from a physical meeting.

## Discussion

As few telerehabilitation interventions have evolved beyond the pilot phase, understanding the determinants of success is key to design better interventions. This paper explored adherence and factors affecting satisfaction in long-term telerehabilitation for patients with COPD from the participants’ perspectives. First, the results suggest that the potential in long-term telerehabilitation over the existing offer of exercise maintenance programs after conventional PR is threefold. Telerehabilitation has the ability to overcome geographical distance. A travel time greater than 30 min is a known barrier for attending PR and exercise programs for patients with COPD [41]. Another advantage of telerehabilitation lies in the specialist access and the potential regularity of follow-up by the same health personnel over a long term. Second, the results identified four key factors to be considered when designing and delivering future telerehabilitation interventions for patients with COPD in order to increase adherence and satisfaction. These key factors are: (i) adherence to different components of the telerehabilitation intervention is dependent on the level of focus provided by the health personnel involved; (ii) the potential for regularity that lies within the technology should be exploited to avoid relapses after vacation; (iii) motivation might be increased by tailoring individual consultations to support experiences of good health and meet individual goals and motivational strategies; (iv) interactive functionalities or gaming tools might provide peer-support, peer-modelling and enhance motivation.

### Adherence

According to the WHO, adherence to long-term therapy in chronic diseases averages to 50 % [34]. In our two-year study, the average adherence rate for diary registrations and training sessions was 43.3 % and 56.2 % respectively. Compared to a nine-month intervention consisting of a web-based home exercise program, an activity coach for ambulant activity registrations and feedback, self-management via a web-based triage diary, and teleconsultations, adherence in the current study was higher for the training sessions, but lower for the web-based diary [16]. Reasons might be that our intervention had a stronger focus on exercise rather than monitoring symptoms and early recognition of exacerbations. A more active education on the recognition of early symptoms of exacerbation and the importance of treatment together with a provision of a straightforward treatment regimen might support the participants’ adherence to fill in the diary. On the other hand, the patients in our study were in a quite stable condition of their disease, and registering symptoms every other day (50 % adherence) throughout a period of two years might be enough to detect early changes in symptoms and start treating exacerbations as soon as possible. An average adherence rate of 56.2 % for the training sessions equals to 1.7 training sessions per week. Compared to recommendations of 3 training sessions per week, this rate is probably too low to maintain the experienced health benefits and increased physical performance. Even though the average adherence rate was kept lower than recommended, we consider the intervention successful as all participants maintained their exercise and self-management routines for the 2-year period. No other studies have succeeded in such a result. However, we believe that while a lower adherence for the diary registrations does not have any negative impact, it is important to focus on patient’s compliance to exercise, which is the key feature of traditional PR programs as well as of the current telerehabilitation intervention.

Adherence rate for training sessions increased during the first three months of the study to a level close to the recommendation. This could be due to a learning effect or an increase in motivation. Adherence was also affected by seasonality. The participants were encouraged to take time-off and did not receive follow-up via videoconferencing from the tele-physiotherapist during holidays. This influenced the lower adherence in these periods. As the participants appreciated the regularity of the telerehabilitation intervention and found it difficult to start exercising again after holidays, we would recommend to exploit the potential for regularity that lies within the technology. Future telerehabilitation interventions could benefit from prescheduled peer-group meetings or exercise sessions via videoconferencing, mobile text message reminders aimed to motivate individual training, online follow-along exercise videos or other gaming elements for exercise training during holidays to avoid relapses.

### Factors affecting satisfaction and adherence, and potential service improvements

Health benefits, experienced in terms of increased physical performance and self-management, were highlighted by the participants as the main factor affecting satisfaction. Despite having a chronic disease, the participants gave an optimistic and varied picture of their definitions of good health. It seems important to foster these positive perspectives of health in long-term telerehabilitation interventions. Good health is often seen as medicine’s main goal [42]. If individual experiences of good health represent a primary objective in long-term telerehabilitation services for patients with COPD, health professionals cannot rely on following up all patients in the same manner. It becomes important to find out what corresponds to the patients’ “own peak” and what good health is for the individual patient. This corresponds well to recent holistic views of health and theories of health promotion [27–29, 42, 43].

During the two-year participation in the pilot study, participants expressed an increase in self-worth and acceptance of their condition. They became more aware of their own needs and how they could fend for themselves. Despite feeling vulnerable, patients with COPD often come to an acceptance of their situation, which lead them to be able to adapt to their physical limitations and make the most out of their situation [44]. Their condition is perceived as a “way of life” rather than an illness, where symptoms and health problems becomes the normality. This “passive acceptance” might hinder patients to address healthcare needs [45]. In contrast, the participants in our study developed a newfound control over their own health situation and a renewed interest in their own health. When taking actions in terms of exercising, they felt they could cope with the vulnerability and stigma from having a chronic and often self-inflicted disease.

Conventional PR is patient-tailored, and core generic strategies such as goal setting, goal assessment, problem solving and decision-making are important elements in self-management for patients with COPD [2]. Patients’ goals and resources are often assessed against their specific context and opportunities in the environment [46]. In our long-term telerehabilitation intervention, there was no formal functionality for goal setting and goal attainment included in the webpage. Maintenance of motivation was perceived by participants as a main challenge. They were motivated to exercise as long as they experienced progress in physical capacity and the exercise program could be progressed. However, motivation decreased once their threshold for progress in physical capacity was reached. This happened for most of the participants after the first year of follow-up. Adherence might benefit if patients with COPD are involved in their own rehabilitation process. An agreement of goals and methods between the patient and the helper is seen as an important key to the change process [30]. Further, patients with COPD might need support to define visible results and appraise their newly acquired competences to be able to implement them in everyday life [12, 47]. A previous qualitative study revealed that patients with COPD expressed and alternated between four attitudes towards telerehabilitation: indifference, learning as part of situations in everyday life, feeling of security, and motivation to perform physical exercise [24]. These experiences with telerehabilitation are to some extent congruent with our findings. In the aforementioned study, indifference was related to measuring stable values (of blood pressure, pulse, weight, spirometry and oxygen saturation). This implied that stable patients with COPD would not benefit as much from telerehabilitation as patients with more unstable conditions. Conventional PR is recommended for all patients with COPD, even patients in earlier stages [2]. Patients in earlier stages are often medically stable, but they would still benefit from rehabilitation in terms of enhanced mastery of everyday life and increased physical performance. Visualisation and documentation of tangible signs of improvements and maintenance might reduce the feeling of indifference and make long-term telerehabilitation suitable and beneficial for patients in stable conditions as well. Future telerehabilitation services, particularly in chronic diseases, should therefore also be patient-tailored, focusing on documentation and evaluation of goals, progress and maintenance. Likewise, it is important to assess individual interpretations of good health and preferences in motivational strategies so that every participant can be followed up according to his or her own needs and values.

### Potential for technological improvements

We have already suggested exploiting the potential for regularity that lies within the technology. Motivational strategy seemed to differ among the participants with adherence above 70 %. We see a potential in using the technology to include tailored motivational messages based on the individuals’ promotion or prevention-goal orientation on the webpage or via mobile text messages [31, 48].

Increased self-efficacy and independence were among the factors affecting satisfaction. Together with increased physical capacity, increased self-efficacy helped the participants in regaining energy to enjoy and participate in social activities, including family life. One of the best ways to increase self-efficacy is through mastery experiences [26]. By provision of equipment for effective exercise training, self-monitoring and supervision in the home environment, the participants seemed to have experienced many positive mastery experiences. In this study, a treadmill and pulse oximeter formed the foundation for new mastery experiences. The best way to provide mastery experiences via telemedicine is not known. Other studies piloted different activity monitors [15, 16].

Self-efficacy can also be created and strengthened through peer-modelling processes or vicarious experiences provided by social models. Seeing people similar to oneself succeed by sustained effort raises the observers’ beliefs that they too can be able to master the opposing challenge [26, 49]. Telemedicine can easily support peer-modelling processes through interactive interaction or gaming tools [15]. Online group exercise sessions for patients with COPD have been piloted successfully [50]. Telemedicine can also facilitate peer-support and regular exchange of experiences that our participants have missed [51]. Group exercise sessions or self-scheduled meetings between the participants supported by videoconferencing or gaming tools might be provided in future telerehabilitation interventions. Peer-support can also be provided through chat rooms or discussion forums where participants can share their experiences [52]. It is known that social support from a group contributes to increased motivation for exercise and exercise maintenance in COPD [44].

### Limitations

As most of the results in this study is based on qualitative data, strengths and weaknesses are assessed against the four criteria for trustworthiness in qualitative research proposed by Guba and discussed by Shenton: credibility; transferability; dependability; and confirmability [53]. All participants were given the opportunity to express their experiences and reflections regarding attendance in the study through a triangulation of methods in a familiar atmosphere. Authors’ immediate understanding of the participants’ answers were summarised consecutively during the focus groups, so that participants’ interpretations were confirmed, thus increasing credibility. We conducted a pilot study with a small number of patients with COPD. Results might therefore not necessarily be transferred to other settings or patient groups. Nevertheless, the findings are somewhat congruent with the existing body of evidence. In terms of dependability, an effort was made to transparently describe the research process and the analysis procedure. The conduction of the two focus groups and the analysis of the material were made by more than one of the authors, thus increasing confirmability. On the other hand, the physiotherapist conducting the videoconferences was also present during the focus groups. The participants might therefore have held back some personal thoughts. However, our overall impression is that triangulation of data collection allowed the participants to provide their critical views as well, thus reducing the effect of investigator bias. Despite being conscious of our hypothesis and theoretical background during data collection and analysis process, these might have affected our interpretations.

During the first year, participants recognised affiliation to the research study as a motivational factor. Hence, adherence and positive experiences perceived during the first year might have been affected by the Hawthorne effect. Being observed might change research participants’ behaviour or answers to what they think is expected [54]. However, only few studies on this concept have demonstrated the Hawthorne effect beyond a 6-months period [54].

No individual interviews were conducted in this study. This is considered a limitation. However, focus groups were chosen to facilitate a creative process among the participants and to open for a thematic analysis of new angles on long-term telerehabilitation from the participants' view. Individual feedback and experiences were provided by individual open-ended questionnaires.

### Implications for research

The adherence rate differed widely among the participants in this pilot study. Our research was not designed to investigate which personal characteristics influence adherence to telerehabilitation. Future studies should implement telerehabilitation interventions on a large-scale and explore how adherence differs among patients, thus producing useful knowledge to develop stratification tools for telerehabilitation interventions.

## Conclusions

No other studies have focused on long-term exercise maintenance via telerehabilitation. The current study suggests that long-term exercise maintenance is feasible and that patients with COPD successfully adhered to the intervention for the long-term duration of 2 years. Our findings revealed some factors that are important for the successful use and acceptance of the intervention. Satisfaction was supported by experienced health benefits, increased self-efficacy and emotional safety. Maintenance of motivation was a challenge and might have affected long-term adherence. Four key factors of potential improvements in long-term telerehabilitation were identified: (i) adherence to different components of the telerehabilitation intervention is dependent on the level of focus provided by the health personnel involved; (ii) the potential for regularity that lies within the technology should be exploited to avoid relapses after vacation; (iii) motivation might be increased by tailoring individual consultations to support experiences of good health and meet individual goals and motivational strategies; (iv) interactive functionalities or gaming tools might provide peer-support, peer-modelling and enhance motivation.

## Abbreviations

COPD: Chronic obstructive pulmonary disease
PR: Pulmonary rehabilitation
SUS: The System Usability Scale
